# Personality disorder coverage, prevalence, and convergence: do the *DSM-5*'s two models of personality disorder identify the same patients?

**DOI:** 10.1017/S0033291724000357

**Published:** 2024-07

**Authors:** Lee Anna Clark, Eunyoe Ro, Hallie Nuzum, Emily N. Vanderbleek, Xia Allen

**Affiliations:** 1 Department of Psychology, University of Notre Dame, Notre Dame, IN, USA; 2 Department of Psychology, Southern Illinois University Edwardsville, Edwardsville, IL, USA; 3 Veterans Affairs Puget Sound Health Care System, Seattle Division, Seattle, WA, USA; 4 James A. Haley Veterans Hospital, Neuropsychology, Tampa, FL, USA; 5 And Still We Rise, LLC, Boston, MA, USA

**Keywords:** Alternative *DSM-5* Model for Personality Disorders, interrater reliability, prevalence, traditional categorical–alternative dimensional PD model convergence, personality disorder classification

## Abstract

**Background:**

Research on the Alternative *DSM-5* Model for Personality Disorders (AMPD) in *DSM-5*'s Section-III has demonstrated acceptable interrater reliability, a largely consistent latent structure, substantial correlations with theoretically and clinically relevant measures, and evidence for incremental concurrent and predictive validity after controlling for *DSM-5*'s Section II categorical personality disorders (PDs). However, the AMPD is not yet widely used clinically. One clinician concern may be caseness – that the new model will diagnose a different set of PD patients from that with which they are familiar. The primary aim of this study is to determine whether this concern is valid, by testing how well the two models converge in terms of prevalence and coverage.

**Method:**

Participants were 305 psychiatric outpatients and 302 community residents not currently in mental-health treatment who scored above threshold on the Iowa Personality Disorder Screen (Langbehn et al., 1999). Participants were administered a semi-structured interview for *DSM-5* PD, which was scored for both Section II and III PDs.

**Results:**

Convergence across the two PD models was variable for specific PDs, *Good* when specific PDs were aggregated, and *Very Good* for ‘any PD.’

**Conclusions:**

Results provide strong evidence that the AMPD yields the same overall prevalence of PD as the current model and, further, identifies largely the same overall population. It also addresses well-known problems of the current model, is more consistent with the *ICD-11* PD model, and provides more complete, individualized characterizations of persons with PD, thereby offering multiple reasons for its implementation in clinical settings.

Problems with *DSM-5*-Section II's traditional, categorical model of personality disorders (PDs) are well known, particularly (a) high levels of comorbidity among PDs and with clinical syndromes, all of which are supposed to be distinct disorders and (b) significant within-PD heterogeneity, which undermines the expectation that each personality disorder is a coherent entity. One major goal of the Alternative *DSM-5* Model for Personality Disorders (AMPD) in Section-III, ‘Emerging Measures and Models,’ was to address these shortcomings. It offers a PD conceptualization with two main elements: Criterion A – impairment in personality functioning (self- and/or interpersonal functioning) – which is expressed via Criterion B – one or more pathological-range personality traits. Both criteria are dimensional, spanning from no impairment and adaptive-range traits to extreme impairment and pathological expression of one or more personality traits.

To provide a bridge from the traditional model, the AMPD also includes six specific PDs: Antisocial, Avoidant, Borderline, Narcissistic, Obsessive-compulsive, and Schizotypal PD. Each is defined by a specific version of moderate or greater impairment in personality functioning and a particular set of traits with a specific number and configuration of traits required for diagnosis. Finally, the AMPD includes PD-Trait Specified (PD-TS) for specification of PD in anyone meeting the general PD criteria. (Note that per the AMPD, PD-TS should be used only when individuals meet the general PD criteria but not the criteria for any specific PD. However, previous research (Clark et al., 2015) has demonstrated the utility of an expanded definition of PD-TS.) Notably, these AMPD diagnoses suffer from the same problems of comorbidity and heterogeneity as those in Section II (see Tables 1 and 2), with the one difference that the reasons for the comorbidity and heterogeneity are more transparent (see Lynam & Widiger, 2001, for an excellent example of how traits explain comorbidity).

**Table 1.** Percentage of comorbid diagnoses between each pair of section II personality disorders

	Diagnosis										
Diagnosis	PAR	SZD	STP	ANT	BOR	HIS	NAR	AVD	DPN	OC	Total
Paranoid PD		13.2	21.1	26.3	**39**.**5**^a^	5.3	26.3	23.7	2.6	15.8	38
Schizoid PD	**41.7**^a^^b^^c^		25.0^c^	25.0	16.7	0.0	8.3	**33.3**	0.0	8.3	12
Schizotypal PD	**47.1**^a^^b^^c^	17.6^c^		23.5	**35.3**	0.0	29.4	17.6	0.0	17.6	17
Antisocial PD	20.0	6.0	8.0		**30.0**^a^	4.0	24.0	18.0	4.0	18.0	50
Borderline PD	25.4	3.4	10.2	25.4		5.1	18.6	**33.9**^a^	6.8	23.7	59
Histrionic PD	25.0	0.0	0.0	25.0	**37.5**		**62.5**^a^^b^^c^	0.0	0.0	12.5	8
Narcissistic PD	**33.3**	3.3	16.7	**40.0**^a^^b^^c^	**36.7**	16.7^c^		6.7	0.0	26.7^c^	30
Avoidant PD	18.4	8.2	6.1	18.4	**40.8**^a^^b^^c^	0.0	4.1		22.4^c^	26.5	49
Dependent PD	5.3	0.0	0.0	10.5	21.1	0.0	0.0	**57.9**^a^^b^^c^		10.5	19
Obssv-Cmplsv PD	12.2	2.0	6.1	18.4	28.6	2.0	16.3	26.5^a^	4.1		49

PAR, paranoid; SZD, schizoid; STP, schizotypal; ANT, antisocial; BOR, borderline; HIS, histrionic; NAR, narcissistic; AVD, avoidant; DEP, dependent; OC, obsessive compulsive; Obssv-Cmplsv, obsessive compulsive.

*Note*: Numbers indicate the percentage of the personality disorder (PD) in that row that is comorbid with the diagnosis in each column. For example, of the 30 individuals diagnosed with narcissistic PD, 16.7 were also diagnosed with schizotypal PD, 40% with antisocial PD, and 26.7% with OCPD. In contrast, of the 17 individuals diagnosed with schizotypal PD, 29.4% were also diagnosed with narcissistic PD, of the 50 individuals diagnosed with antisocial PD, 24% were also diagnosed with narcissistic PD, and of the 49 individuals diagnosed with OCPD, 16.3% were also diagnosed with narcissistic PD.

Percentages ⩾30% are in **bold**; those between 20% and 29% are underlined.

a Highest percentage of comorbidity in each row.

b Indicates pairs of PD diagnoses that tend to be specifically comorbid with each other.

c Highest percentage of comorbidity in each column.

**Table 2.** Percentage of comorbid diagnoses between each pair of section-III (AMPD) personality disorders

	PD diagnosis										
Diagnosis	ANT	AVD	BOR	NAR	OC	STP	PAR	SZD	HIS	DPN	Total
Antisocial PD		0.0	**41.2**^a^	**41.2**^a^^b^^c^	0.0	5.9	**70.6**	11.8	17.7	0.0	17
Avoidant PD	0.0		**36.8**^a^	10.5	18.4	**34.2**^c^	**63.2**	**55.3**	5.3	21.1	38
Borderline PD	8.3	16.7		13.1	4.8	21.4^a^	**66.7**	15.5	20.2	19.1	84
Narcissistic PD	21.2	12.1	**33.3**^a^		9.1	15.2	**66.7**	12.1	**51.5**	6.1	33
Obssv-Cmplsv PD	0.0	**38.9**^a^^b^^c^	22.2	16.7		27.8	**50.0**	**50.0**	11.1	16.7	18
Schizotypal PD	2.8	**36.1**	**50.0**^a^^b^^c^	13.9	**41.2**^c^		**94.4**	**33.3**	13.9	5.6	36
Paranoid PD^d^	10.3	20.7	**48.3**	19.0	7.8	29.3		21.6	12.9	11.2	116
Schizoid PD^d^	4.9	**51.2**	**31.7**	9.8	22.0	29.3	**61.1**		4.9	9.8	41
Histrionic PD^d^	10.3	6.9	**58.6**	**58.6**	6.9	17.2	**51.7**	6.9		13.8	29
Dependent PD^d^	0.0	25.0	**50.0**	6.3	9.4	6.3	**40.6**	12.5	12.5		32

PAR, paranoid; SZD, schizoid; STP, schizotypal; ANT, antisocial; BOR, borderline; HIS, histrionic; NAR, narcissistic; AVD, avoidant; DEP, dependent; OC, obsessive compulsive; Obssv-Cmplsv, obsessive compulsive.

*Note:* Numbers indicate the percentage of the personality disorder (PD) in that row that is comorbid with the diagnosis in each column. For example, of the 33 individuals diagnosed with narcissistic PD, 33.3% were also diagnosed with borderline PD, whereas of the 84 individuals diagnosed with borderline PD, 13.1% were also diagnosed with narcissistic PD.

Percentages ⩾30% are in **bold**; those between 20% and 29% are underlined. For the six specific AMPDs.

a Highest percentage of comorbidity in each row.

b Indicates pairs of PD diagnoses that tend to be specifically comorbid with each other.

c Highest percentage of comorbidity in each column.

d PDs not specified in the AMPD. These were scored using trait facets identified tentatively by the *DSM-5* Personality and Personality Disorder Work Group, as follows. Paranoid PD – two or more of suspiciousness, unusual beliefs and experiences, and hostility; schizoid PD – three or more of withdrawal, intimacy avoidance, anhedonia, and restricted affectivity; histrionic PD – emotional lability and attention seeking; dependent PD – separation insecurity and submissiveness. As with the primary six AM-PDs, the selected traits were required to be in the pathological range – 2 or 3 of a 0–3 rating scale. However, because the trait selections for these PDs were never validated, these results are preliminary and results with these variables must be considered with caution.

Regarding comorbidity, the AM-PDs all have Criterion A in common and the Criterion B traits shared by pairs of PDs are a known source of overlap. In contrast, except for a few cases in which Section-II PDs have a criterion in common (e.g. Schizoid and Schizotypal PD share the criterion ‘Lacks close friends or confidants other than first-degree relatives’), the reason for comorbidity between Section-II PDs is less clear. Regarding heterogeneity, the average internal consistency reliability (Cronbach's *α*) of the Section-II PDs in the study sample is 0.67, with an average within-PD criterion *r* of 0.20. The AM-PDs fare little better: Their average *α* is also 0.67, with only a slightly higher average within-PD trait *r* of 0.34.

The difference between the models is again transparency: The Section-II PDs are presumed to be coherent entities, so the fact that they are only moderately so is problematic. In the AMPD, however, variation across the six PDs' consistency *v.* heterogeneity is based in their component facets. For example, Antisocial PD may have the highest *α* (0.86) and among the higher average trait-criterion *r*s (0.46) because its six trait components are three facets from each of two domains (Antagonism and Disinhibition) that are themselves correlated (*r* = 0.61). In contrast, Borderline PD may be more heterogeneous (i.e. its *α* = 0.75 and average trait-criterion *r* = 0.30, somewhat lower than Antisocial PD's) because five of its components come from three different domains (APA, 2013; Clark & Watson, 2022). Accordingly, when individuals are diagnosed with AMPD Borderline PD, heterogeneity among them may be explained by reference to their trait profiles. Similarly, Section II PD heterogeneity could be explained by reference to which diagnostic criteria are met, but this is not customary practice, perhaps because it requires assessing all 79 diagnostic criteria (i.e. all PD criteria contribute to heterogeneity, not just the focal PD's).

Considerable published research on the AMPD exists, with a recent review of over 200 articles demonstrating ‘(a) acceptable interrater reliability, (b) largely consistent latent structures, (c) substantial correlations with a range of theoretically and clinically relevant external measures, and (d) some evidence for incremental validity when controlling for categorical PD diagnoses’ (Zimmermann, Kerber, Rek, Hopwood, & Krueger, 2019, p. 91).

Further, a recent ‘Ten-Year Retrospective’ Special Issue of *Personality Disorders: Theory, Research, and Treatment* included four reviews of, respectively, Criterion A, Criterion B, the two together, and AMPD's clinical utility, each with three commentaries, plus reviews of its specific PDs. Despite considerable diversity in perspective across papers, the journal's editors (Sharp & Miller, 2022) noted convergence on several views of the model, including that ‘no information is lost by the abolishment of the six categories as they are well represented by Criterion A and Criterion B features’ (p. 303).

Nevertheless, the AMPD still represents a minority of published PD research, with borderline PD (BPD) garnering almost half (47%) of the recently published PD literature (Watson & Clark, 2023). Similarly, BPD accounted for 48% of talks at the 2022 conference of the North American Society for the Study of Personality Disorders. Given the literature showing the correlational convergence of the Section II and III PDs (e.g., Miller, Few, Lynam, & MacKillop, 2015, Table 3 reports a mean convergent *r* of 0.63 and a median discriminant *r* of 0.25 between criterion counts of Section-II PDs and trait counts of the Section-III [AMPD] PDs; see online Supplementary Table S1 for parallel results in our dataset), the predictive power of AMPD traits for the Section-II PDs (e.g., Bastiaens, Smits, De Hert, Vanwalleghem, & Claes, 2016), evidence that the AMPD taps the same genetic risk factors as the Section-II PDs (Reichborn-Kjennerud et al., 2017), and the similarity in their predictive power for external correlates (Miller et al., 2015), why has the PD research field not embraced the AMPD? Further, there is very little evidence that the AMPD is being used clinically, except perhaps in clinical psychology training clinics, raising the question, ‘Why not?’

**Table 3.** Interrater reliability ratings – components of personality disorder diagnoses

		ICCs	
Measure	Kappa (0–1)	Fully Dimensional	# High Components
Section-II personality disorders –general PD criteria			
Criterion A (enduring pattern)	**0.72**	—	0.70
All general PD criteria	**0.73**	—	—
Alternative model of personality disorder – criteria A and B			
Criterion A rated^a^	**0.63**	—	—
Criterion A scored from components^b^	0.59	**0.76**	0.65
Criterion B rated^c^	0.50	—	—
Criterion B scored from components^d^	*0*.*39*	**0**.**83**	0.73
All general PD criteria	0.55	—	*0.55*
Domains and primary facets			
**Negative affectivity (NA)**	**0.73**	**0.78**	0.71
Emotional lability	0.49	0.65	—
Anxiousness	0.51	0.60	—
Separation insecurity	**0.87**	**0.82**	—
**Detachment (DET)**	**0.73**	**0.78**	0.71
Withdrawal	0.55	0.74	—
Intimacy avoidance	0.43	0.60	—
Anhedonia	**0.74**	0.64	—
**Antagonism (ANT)**	0.56	**0.81**	**0.79**
Manipulativeness	**0.71**	0.74	—
Deceitfulness	*0*.*39*	0.50	—
Grandiosity	**0.74**	0.68	—
**Disinhibition (DIS)**	0.54	0.70	*0.59*
Irresponsibility	**0.72**	0.80	—
Impulsivity	0.59	0.74	—
Distractibility	*0*.*21*	0.51	—
**Psychoticism (PSY)**	0.54	0.70	*0.59*
Unusual beliefs & experiences	0.57	0.57	—
Eccentricity	—^e^	0.30	—
Cognitive & perceptual distortion	0.56	0.65	—
Secondary facets (Domain)			
Restricted affectivity (DET)	—^e^	0.21^f^	—
Depressivity (DET, NA)	0.56	0.70	—
Attention seeking (ANT)	**0.81**	**0.77**	—
Callousness (ANT)	**0.64**	0.62	—
Hostility (ANT)	**0.67**	0.68	—
Risk taking (DIS)	**0.64**	0.63	—
Perseveration (NA)	**0.62**	0.65	—
Rigid perfectionism (NA)	*0.23*^f^	0.23^f^	—
Other facets			
Submissiveness	0.57	0.66	—
Suspiciousness	0.49	0.68	—

*Note:* ICC = intraclass correlation (Shrout & Fleiss, ICC[1,1]). Kappa ranges are those used in the *DSM-5* field trials (Regier et al., 2013): Kappa **= 0.60–0.79 (Very Good, bolded)**; kappa = 0.40**–**0.59 (Good, underlined); *Fair* *=* *0.20***–***0.39* (*italicized*); Poor ⩽0.20. **ICC** – **Excellent ⩾0.90**, **Very good** = **0.75–0.89**, Good = 0.60–0.74, *Fair* = *0.40–0.59*, Poor⩽0.40. — = Not applicable. **Domain names** are **bolded**.

a Rated 0–4; ratings ⩾2 indicate moderate or greater impairment overall and for each of four components.

b To meet Criterion A by scoring, at least two components (Identity, Self-direction, Empathy, Intimacy) must be rated ⩾2; Fully Dimensional = summed score over all four components; # High Components = for Criterion A, number of four components rated ⩾2; for Criterion B traits, number of facets rated ⩾2.

c Rated 0–3; ratings ⩾2 indicate pathological range overall and for all domains and facets.

d To meet Criterion B by scoring, one or more facets or domains must be rated ⩾2; Fully Dimensional = summed score over all facets; # High facets = number of 25 facets and domains rated ⩾2.

e Too few positive cases in interrater reliability subsample to calculate reliably.

f Values may be unreliable due to inconsistency across raters re: rating the constructs' high or low end (see text for details).

We posit that several factors jointly act as barriers to clinical use of the AMPD, at least in the U.S. First, the U.S.'s official diagnostic system for reporting health data and used by third-party payers is the *ICD-10-Clinical Modification* (*ICD-10-CM*), which is based on *DSM-5*, Section II. In contrast, the *International Classification of Diseases, 11^th^ Edition (ICD-11)* became the diagnostic system for use worldwide with its ratification by the World Health Organization in 2019 and required implementation in 2022. The *ICD-11* PD model is fully dimensional, requiring only a severity level of impairment (mild, moderate, or severe), and providing optional trait specifiers for further characterization. A second factor inhibiting the AMPD's clinical use concerns its assessment, both of Criterion A and B. Regarding Criterion B, there may be among clinicians a lack of familiarity with multi-dimensional trait models. The Personality Inventory for *DSM-5* (PID-5; Krueger, Derringer, Markon, Watson, & Skodol, 2012), the AMPD's official self-report measure, is freely available from the American Psychiatric Association (https://www.psychiatry.org/psychiatrists/practice/dsm/educational-resources/assessment-measures) and is the most commonly used Criterion B measure. However, measures that require formal scoring of multiple scales are rarely used in clinical practice, perhaps because the field lacks the infrastructure needed to score and interpret them.

Regarding Criterion A, the official Level of Personality Functioning Scale (LPFS) is a set of descriptors within the *DSM-5*. Semi-structured interviews to assess it exist (Bender, Skodol, First, & Oldham, 2018; Hutsebaut, Kamphuis, Feenstra, Weekers, & De Saeger, 2017; Thylstrup et al., 2016), as do numerous self-report measures, including three developed pre-*DSM-5* (i.e. Livesley, 2010; Parker et al., 2004; Verheul et al., 2008), which contributed to the LPFS's development (Morey et al., 2011), and an increasing number developed post-*DSM-5* (e.g., Gamache, Savard, Leclerc, & Côté, 2019; Huprich et al., 2018; Hutsebaut, Feenstra, & Kamphuis, 2016; Morey, 2017; Weekers, Hutsebaut, & Kamphuis, 2019)*.* For details regarding and/or reviews of these measures, see Birkhölzer, Schmeck, & Goth, 2021; McCabe, Oltmanns, & Widiger, 2021; Morey, McCredie, Bender, & Skodol, 2022; Sharp & Wall, 2021; Waugh et al., 2021; Zimmermann et al., 2020). However, semi-structured interviews require training to ensure reliable and valid use, and existing self-report measures, like the Criterion B trait measures, require scoring multiple scales, which together may inhibit the AMPD's clinical use.

A third factor in the slow AMPD uptake by U.S. clinicians may concern caseness – that the current set of PD patients will not meet the AMPD's criteria and that other types of patients will. This concern subsumes convergence on both prevalence and coverage (e.g., whether the size and nature of the patient population currently diagnosed with PD will change using the AMPD *v.* the traditional model).

This paper aims primarily to address the third of these factors: To determine the extent to which – and the ways in which – *DSM-5*'s Section-II categorical model and the AMPD converge *v.* differ in terms of caseness – PD prevalence and coverage. We used a semi-structured interview to examine these factors across models.

## Method

### Participants

Participants were recruited from a medium-sized Midwestern U. S. city and surrounds via two sources: (1) psychiatric outpatients referred primarily from a community mental health center and (2) community residents not currently in mental-health treatment but above threshold on the Iowa Personality Disorder Screen (IPDS; Langbehn et al., 1999). We combined these subsamples for our primary analyses (presented in the section ‘Convergence Between Models)’ to allow us to examine our key questions about personality disorder (PD) assessment in a sample that overall reflected the actual circumstances in which clinicians conduct a PD diagnostic assessment. Specifically, clinicians often (1) are referred patients with various clinical diagnoses and asked to comment on whether the patients' clinical presentation also reflects PD; and (2) take on new clients who describe intrapsychic and/or interpersonal difficulties that suggest PD, which we mimicked by screening-in community adults with high PD risk. The former were referred by mental-health care providers who had agreed to provide patients – both new patients and those in ongoing treatment – with a brief description of the study if they deemed that the patients met the study requirements (see below). Patients then contacted the lab if they were interested in participating.

The community residents were contacted via random selection of landlines and cell phones in the targeted area by a social-science research center and screened by the center for study eligibility, including the following, as well as begin above threshold on the IPDS. Study requirements were that participants had to be at least 18 years old, able to complete self-report questionnaires and interviews in English, and absent delirium, dementia, or active psychotic symptoms. Of the total sample (*N* = 612), 302 community residents and 305 outpatients completed the measures used in this study.

Mean age was 46 (*s.d.* = 13); the sample was 57% female; 68% white, 21% black, and 11% mixed race/other. There were no sample differences on age or sex; however, the patient sample had a higher percentage of non-white individuals (25% *v.* 18% black; 13 *v.* 7% mixed/race other). To characterize the overall sample and subsamples further, the frequency of diagnoses of 13 common clinical disorders (e.g., MDD, GAD, OCD, PTSD, SUD) in the overall sample and each subsample is provided in Supplemental Table S2. For eight of the diagnoses either the base rate and/or severity was higher in patients. The frequency of Section II and AM-PD diagnoses overall and by subsample is provided in Supplemental Table S3.

### Procedure

Participants came to the research lab and, after having reviewed a description of the research and having their questions, if any, answered, provided written informed consent, and then completed both computerized self-report questionnaires and interviews. Participants completed the study in 1 day or across 2 days per their availability and preference. They were provided breaks with food/beverages to promote engagement and help prevent fatigue.

The authors assert that all procedures contributing to this work comply with the ethical standards of the University of Notre Dame Institutional Review Board overseeing human subjects research, protocol #17-12-4289.

### Measures

#### Structured interview for DSM personality

The SIDP (Pfohl, Blum, & Zimmerman, 1997) is a semi-structured interview that assesses respondents' emotions, cognitions/attitudes, and behaviors across various situations, focused on respondents' ‘usual self.’ Interviewers rated the General PD Criteria (e.g., ‘inflexible and pervasive,’ ‘stable and of long duration’) dichotomously and rated each *DSM-5* Section-II criterion on a 0–3 scale (0 = minimal/not present; 1 = present below diagnostic threshold; 2 = present at/above diagnostic threshold; 3 = prominent manifestation of personality pathology). They then rated the AMPD, including its General PD Criteria, which are largely the same as Section II's, rating Criterion A both dichotomously (i.e. no/minimal *v.* moderate or greater personality-functioning impairment) and via its four subcomponents – identity, self-direction, empathy, and intimacy – using the LPFS's scale: 0–4 = little to no, some, moderate, severe, or extreme impairment. They rated Criterion B's five trait domains – Negative Affectivity, Detachment, Antagonism, Disinhibition, and Psychoticism – and 25 facets using a 0–3 rating scale – very little/not at all, mildly, moderately, or extremely descriptive. Descriptive statistics (*Mean*, *s.d.*, range, Cronbach's *α*, McDonald's *ω*) for all study variables are provided in Supplemental Table S5.

*Interrater Reliability*. Interviewers completed rigorous training and thereafter met weekly to discuss questions regarding ratings, which were resolved by consensus. Midway through, and again after data collection was completed, a subset of each rater's interviews was selected and assigned randomly for re-rating by another interviewer; no two raters were paired more than twice. A total of 13 interviewers (two were Ph.D.-level psychologists, one had a master's degree in a related field, two had bachelor's degrees in psychology and the remaining eight were advanced graduate students in clinical psychology; virtually all interviewers also served as re-raters) re-rated 54 interviews using audiotaped recordings. Interrater reliability coefficients were calculated – kappa (*κ*) for categorical ratings and intraclass correlations (ICC; using Shrout and Fleiss, 1979, formula [1,1]) for dimensional ratings.

## Results

### Preliminary analyses

Before comparing the two models, we examined AMPD Criterion A ratings *v.* scores, interrater reliability, comorbidity in the models separately, and coverage by specific PDs *v.* AMPD Criterion A. Criterion A base rate was 48.3% when *rated* overall, 38.1% when *scored* using its subdomains, and 48.9% considering either. Only five participants who met the *scored* threshold were not *rated* as impaired. Accordingly, we allowed Criterion A to be met by either method for all AMPD diagnoses reported herein.

#### Interrater reliability

We calculated interrater reliabilities for both the categorical and alternative PD models for both their constituent components (Table 3) and diagnoses (Table 4). General PD Section-II Criterion A (‘An enduring pattern of inner experience and behavior that deviates markedly from the expectations of the individual's culture’ [APA, 2013, p. 645]) was scored based on ratings of its four constituent areas: cognition, affectivity, interpersonal functioning, and impulse control. Kappas for whether participants met Section-II Criterion A and all General PD criteria, respectively, were 0.72 and 0.73 (*Very Good;* all interpretations of kappa values reflect the ranges used in the *DSM-5* field trials [Regier et al., 2013]).

**Table 4.** Interrater reliability – section II personality disorder diagnoses and AMPD diagnoses

		ICCs		
Measure		Kappa (0–1)	Fully Dimensional	Categorical (# criteria met)
*DSM-5*, II	Any PD	**0.73**	—	—
*DSM-5*, II	Number of PDs	—	0.71	—
*DSM-5*, II	Paranoid PD	**0.63**	**0.82**	0.72
*DSM-5*, II	Schizoid PD	—^a^	**0.75**	**0.85**
*DSM-5*, II	Schizotypal PD	**0.66**	0.74	**0.78**
*DSM-5*, II	Antisocial PD	0.50	**0.81**	**0.77**
*DSM-5*, II	Borderline PD	**0.70**	**0.84**	**0.85**
*DSM-5*, II	Histrionic PD	**0.66**	0.74	**0.87**
*DSM-5*, II	Narcissistic PD	**0.79**	**0.90**	**0.93**
*DSM-5*, II	Avoidant PD	**0.68**	**0.89**	**0.96**
*DSM-5*, II	Dependent PD^a^	—^a^	**0.87**	0.68
*DSM-5*, II	Obsessive–compulsive PD	—^a^	0.74	**0.83**
AMPD	Any PD	**0.67**	—	—
AMPD	# of PDs per domains	—	0.49	—
AMPD	# of PDs per facets	—	0.62	—
Diagnosis per facets^b^				
PD trait-specified		0.50	—	—
Diagnosis per domains^c^				
AMPD	Avoidant PD	*0.26*	—	—
AMPD	Narcissistic PD	0.56	—	—
AMPD	Obsessive–compulsive PD	0.15	—	—
AMPD	PD trait-specified	*0.30*	—	—

AMPD, alternative (*DSM-5*) model for personality disorder; ICC, intraclass coefficient.

— = not applicable. Fully dimensional = sum of the 0–3 ratings of each Section-II PD's criteria.

Kappa ranges are those used in the *DSM-5* field trials (Regier et al., 2013): Kappa **= 0.60–0.79 (Very Good, bolded)**; kappa = 0.40**–**0.59 (Good, underlined); *Fair* *=* *0.20***–***0.39* (*italicized*); Poor ⩽0.20.

**ICC**: **Excellent ⩾0.90**, **Very good** = **0.75–0.89**, Good = 0.60**–**0.74, *Fair* = *0.40***–***0.59*, Poor <0.40.

a Too few positive cases in the interrater reliability subsample to calculate kappa reliably.

b All specific PDs had too few positive cases diagnosed using their constituent facets in the interrater reliability subsample to calculate kappa reliably.

c Antisocial, borderline, and schizotypal PDs had too few positive cases diagnosed using their constituent domains in the interrater reliability subsample to calculate kappa reliably.

Reliability of AMPD Criterion A (personality impairment) when *rated* overall by interviewers was slightly higher than when *scored* from the four subdomains' ratings (*κ* = 0.63 *v.* 0.59), but both were near the standard cut point of 0.60 between *Good* and *Very Good*. Criterion B was rated overall and scored somewhat less reliably (*κ*s = 0.50 and 0.39, *Good* and *Fair*, respectively). However, the reliabilities (ICCs) of overall trait severity ratings and number of pathological traits were 0.83 and.73 – *Very Good* and *Good*, respectively. This pattern may be attributable to Criterion B's definition, which requires only ‘one or more pathological personality traits’ of five domains and 25 facets. Thus, ratings below threshold for all traits *v.* all but one trait is a ‘miss’ when calculating *κ*, but very close when calculating ICCs.

Most (83%) reliabilities – both *κ*s and ICCs – of Criterion B domains and facets were *Good* to *Very Good*; median *κ*s were *Good* for both domains and primary facets and *Very Good* for non-primary facets. Median ICCs for fully dimensional domain scores were also *Very Good*, and *Good* for all facets. Restricted Affectivity and Eccentricity had too few positive cases in the interrater reliability subsample to calculate *κ* and yielded *Poor* ICCs. Restricted Affectivity's and Rigid Perfectionism's unreliabilities (*Fair κ*; *Poor* ICC) may have resulted from inconsistency across raters regarding rating the constructs' high or low end. These constructs are *labelled* by their high ends but *scored* at the low ends of their respective domains. Thus, some raters may have rated them per their labels as instructed and others per their scoring in the AMPD. In sum, interrater reliabilities of component ratings were *Good* to *Very Good* for both PD models, with a few exceptions for AMPD components.

As for diagnoses (Table 4), for the seven specific Section-II PDs with sufficient cases, *κ* ranged from *Good* to *Excellent*. Mean reliabilities were all in the *Very Good* range: They were highest for fully dimensional scoring (sum of 0–3 ratings of each PD's criteria; ICC*_M_* = 0.85), followed by criterion counts (number of criteria met; ICC*_M_* = 0.81) and then dichotomous diagnoses (*κ_M_* = 0.67). Agreement on the presence of ‘Any PD’ was *Very Good* for both traditional and AMPD diagnoses (*κ* = 0.73 and 0.67, respectively), as was agreement on the number (of 10) PDs for traditional diagnoses (ICC = 0.71) and for the number (of six) AMPD diagnoses based on facets (ICC = 0.62). When based on domains, ICC for number of AMPD diagnoses (0.49) was *Fair*. Calculation of the six specific AMPDs was not possible for facets, due to too-low base rates in the interrater-reliability subsample. When based on domains, *κ*s could be calculated for only Narcissistic, Avoidant, and Obsessive-Compulsive PDs, plus PD Trait-Specified (TS). Only PD-TS and Narcissistic PD had *Good* reliabilities; the others were *Fair* to *Poor*. In sum, kappas for both PD models were *Very Good* overall and *Very Good* for specific Section-II PDs, whereas diagnoses of specific AM-PDs were too infrequent to calculate reliabilities.

#### Comorbidity

Consistent with the literature, of the 202 individuals who met criteria for any Section-II PD, 45% met criteria for more than one PD. Most (83.5%) met criteria for 2 or 3 PDs, with 16.5% meeting criteria for 4 to 6 PDs. In addition, 5% of the sample met SIDP criteria for Mixed PD, defined as not meeting full criteria for any Section-II PD and falling one criterion short of meeting criteria for at least two Section-II PDs. The mean percentage of comorbidity across all pairs of diagnoses was 16.8%; range = 0.0 to 62.5%. See Table 1 for specific data and Supplemental Tables S4a and S4b for the data separately by subsample.

In the AMPD, of the 153 individuals who met criteria for any of the six specific AMPD diagnoses, 33% met criteria for more than one PD. Most (90.4%) met criteria for 2 or 3 PDs, with <1% meeting criteria for 4 or more PDs. The mean percentage of comorbidity across pairs of the six specific AMPD diagnoses was 19.8%; range = 0.0 to 50%, and across all 10 Section II AM-PDs the percentage was 24.1%; range = 0.00 to 94.4%. See Table 2 for specific data and Supplemental Tables S4c and S4d for the data separately by subsample.

Thus, in both models, focusing on any specific PD alone does not provide a complete, accurate diagnostic picture either in general or for a considerable percentage of individuals who receive any PD diagnosis. Moreover, as noted earlier, the AMPD hybrid model does not address the problem of PD comorbidity ipso fact – the AMPD's specific PDs are as or more likely to be comorbid as those of Section II, reflecting the complex nature of PD as a whole.

#### PD coverage by specific PDs

When considering Section-II PDs, the focus is typically on the 10 specific PDs. However, it is well known that the single most prevalent PD is not one of the 10, but PD-not elsewhere classified (PD-NEC, previously PD-not otherwise specified; PD-NOS; Verheul & Widiger, 2004). Similarly, the AMPD is a hybrid model – with dimensions of personality functioning impairment and pathological personality traits used to define six specific PD categories – to provide a bridge from the Section-II PDs to the AMPD's dimensional PD diagnoses to indicate how the AMPD relates to the model in Section II. Nonetheless, a feature of the AMPD is the introduction of PD-TS, which allows trait specification of any PD, not only the six common to the AMPD and Section II. Thus, we next examined the base rates of (1) the 10 Section-II PDs in the aggregate *v.* any Section-II PD (i.e. the 10 specific PDs plus PD-NEC/PD-NOS) and (2) the six specific AM-PDs *v.* any AM-PD (i.e. the six specific AM-PDs plus PD-TS). Individuals were diagnosed with PD-NEC/PD-NOS or PD-TS if they met the General Criteria for a PD in each model, respectively, but did not meet criteria for any of the model's specific PDs, respectively.

Taken together, 202 individuals (33.3% of the sample) met criteria for one or more of the 10 Section-II PDs, whereas 331 individuals (54.5% of the sample) met criteria for one or more of the 10-Section-II PDs or PD-NEC/PD-NOS. Thus, the former group represented only 61% (i.e. 33.3%/54.5%) of the latter, with the individuals diagnosed only with PD-NEC/PD-NOS adding 21.25 percentage points to those with standard PD diagnoses. Similarly, adding PD-TS to the AMPDs six specific PDs yielded almost twice as many PD diagnoses as when only the six specific PDs were diagnosed (48.9% *v.* 25.2%), a base-rate difference of 23.7 percentage points. These data make clear that to characterize PD fully in each system, individuals with any form of personality pathology must be included, not only those who meet criteria for the 10 (Section II) or six (Section-III/AMPD) specific PDs, respectively. Accordingly, we first present data on the two models as they are typically used (i.e. focused on specific PDs), and then examine the complete models, including the ~21–24% of PD diagnoses that is often ignored or mentioned only in passing.

### Convergence between models

We first compared the aggregated prevalence and convergence of the 10 specific PDs in Section II in both models – the 10 Section-II PDs to the six specific PDs of the AMPD, and the two sets of those six PDs (see Table 5). The four PDs not specified in the AMPD were scored using trait facets required to be in the pathological range – 2–3 of a 0–3 rating scale – identified by the DSM-5 personality and personality disorder work group [P and PD WG]. Paranoid PD: two or more of suspiciousness, unusual beliefs and experiences, and hostility; Schizoid PD: three or more of withdrawal, intimacy avoidance, anhedonia, and restricted affectivity; Histrionic PD: emotional lability and attention seeking; Dependent PD: separation insecurity and submissiveness. Because the traits for these diagnoses were only tentatively selected by the P and PD WG and never validated, results with these variables must be considered with caution. In all three comparisons convergence was *Good* (*κ*s = 0.58, 0.55, and 0.57, respectively). Base rates were almost identical (33.28% *v.* 32.29%) in the all-10 PDs comparison; the AMPD's base rate was 8.07% lower than Section-II's when comparing the actual models (10 *v.* six PDs), and 8.40% lower in the 6 PDs comparison. The four omitted PDs represented 10.89% and 21.9% of all 10 PDs in the Section II model and AMPD, respectively.

**Table 5.** Base rates and convergence of section II and section-III (AMPD) personality disorders

	Section II		Section III (AMPD)				
Variable	*n*	%	*n*	%	Base rate Δ	Kappa	(range)^a^
Any PD of 10 specific PDs	202	33.28	196	32.29	0.99	0.58	G
Any 10 Sec. II PD *v.* 6 AM PDs	202	33.28	153	25.21	8.07	0.55	G
Any of 6 AM PDs per Sec. II *v.* III	180	29.65	153	25.21	4.40	0.57	G
Schizotypal PD	17	2.80	36	5.93	-3.13	0.53	G
Antisocial PD	50	8.24	17	2.80	5.45	0.45	G
Borderline PD	59	9.72	84	13.84	-4.12	0.51	G
Narcissistic PD	30	4.94	33	5.44	0.18	**0.66**	VG
Avoidant PD	71	11.70	38	6.26	5.44	*0*.*36*	*F*
Obsessive–compulsive PD	49	8.07	18	2.97	2.10	*0*.*24*	*F*
Dependent PD^b^	19	3.13	32	5.27	-2.14	0.58	G
Paranoid PD^b^	38	6.26	116	19.11	-12.85	*0*.*27*	*F*
Schizoid PD^b^	12	1.98	41	6.75	-4.77	*0*.*34*	*F*
Histrionic PD^b^	8	1.32	29	4.78	-3.46	*0*.*32*	*F*
Any PD	331	54.53	297	48.93	5.3	**0.74**	**VG**

*Note*: *N* = 607. *n* = number of individuals who are positive for the variable in the first column (e.g. 71 individuals met the Section II criteria for Avoidant PD and 38 individuals met the AMPD criteria for Avoidant PD). % = the variables' base rates (i.e. the percentage of the sample that met criteria for the variable in the first column, e.g. 71 of 607 individuals = 11.70%). Base rate Δ = the difference between the base rates for the variable using the Section-II *v.* Section-III (AMPD) criteria (e.g. the difference between 11.70% and 6.26% is 5.44%). Kappa = level of agreement between the Section-II and Section-III (AMPD) diagnosis of the variable in the first column.

a Ranges are those used in the *DSM-5* field trials (Regier et al., 2013): Kappa **= 0.60–0.79 (Very Good, bolded)**; kappa = 0.40**–**0.59 (Good, underlined);  *=* *0.20***–***0.39* (*Fair*, *italicized*); Poor ⩽0.20.

b PDs not specified in the AMPD. These were scored using trait facets required to be in the pathological range – 2–3 of a 0–3 rating scale – identified tentatively by the *DSM-5* Personality and Personality Disorder Work Group, as follows. Paranoid PD: two or more of suspiciousness, unusual beliefs and experiences, and hostility; Schizoid PD: three or more of withdrawal, intimacy avoidance, anhedonia, and restricted affectivity; Histrionic PD: emotional liability and attention seeking; Dependent PD: separation insecurity and submissiveness. As with the primary six AM-PDs, the selected traits were required to be in the pathological range – rated 2 or 3 on a 0–3 scale. However, because the trait selections for these PDs were never validated, the results with these variables are preliminary and must be considered with caution.

Next, we compared each of the specific PDs individually, first the six PDs that are common to both systems and then the four PDs that were specified only tentatively by the Personality and Personality Disorders Workgroup. Base rates for the six PDs in common ranged from 2.8% to 11.7% (*M* = 8.15%) in Section II and from 2.8%–13.8% (*M* = 5.88%) the AMPD. Base-rate differences ranged from -5.5%–12.7% (*M* = 2.6%) and convergence (*κ*) ranged from 0.24–0.66 (*M* = 0.41). Half the convergences were *Fair*, and the rest *Good*, with Narcissistic PD *Very Good*.

Finally, we examined the overall prevalence and convergence of meeting criteria for *any* PD in each system (i.e. including PD-NES/PD-NOS in Section-II and PD-TS in the AMPD). Base rates were very similar across models (54.5% *v.* 48.9%, respectively), with only 9% of participants meeting criteria for a Section-II but not -III (AMPD) diagnosis, and only 4% vice versa. Notably, kappa was 0.74 (*Very Good*).

To summarize, when the 10 (or six) specific PDs were aggregated, convergence (*κ*) was 0.55–.58 (*Good*); when specific PDs were compared individually, roughly half had *Fair* and half *Good* or *Very Good* (one PD) convergence; and when the full models were compared, *coverage* was the highest of any comparison (~50%), *prevalence* was quite comparable across the two models (only a 5.3 percentage point difference), and *convergence* was the highest of all comparisons (*κ* = 0.74).

## Discussion

### Summary

We first showed that the AMPD can be rated reliably (with a few exceptions; see Tables 3 and 4) using a semi-structured interview that was developed to assess the *DSM-5* Section-II PDs. We then presented three different types of evidence that call into question the utility of both the 10 specific PDs in Section II *and* the six in the AMPD: (1) Comorbidity (Tables 1 and 2): Specific PD diagnoses alone do not provide a complete, accurate diagnostic picture either overall or for a considerable percentage of individuals who receive any PD diagnosis. (2) Focusing only on specific PDs means ignoring 20–25% of individuals who can be diagnosed with PD (i.e. meet the General PD Criteria) but do not meet the criteria for a specific PD; (3) Prevalence convergence between the two models is (a) *Fair* (*κ*s = 0.24–0.36) for two of the specific PDs in common, (b) *Good* (*κ*s = 0.45–0.66) for the other four; and (c) *Good* (*κ*s = 0.55–0.58) when the specific PDs are compared in groups (e.g. the six specific PDs of the AMPD in both models). In contrast, convergence is best – *Very Good* (*κ* = 0.74) – when the prevalence of any PD (i.e. including PD-NEC/PD-NOS and PD-TS) is compared across models (Table 5).

### Implications

These results provide insight into how the AMPD may be improved, particularly regarding the hybrid model. First, whether examining a given PD or PDs overall, interrater reliability was weakest for dichotomous diagnoses, and strongest for fully dimensional specification of Section-II diagnoses and AMPD personality functioning impairment and traits. This was particularly true for the AMPD diagnoses because of the relatively small number of traits used to diagnose each PD (*M* = 5), ranging from two (Narcissistic PD) to seven (Antisocial and Borderline PDs).

Second, convergence between the Section II and III/AMPD models was weakest for the specific PDs, ranging from *Fair* to *Good*, at best, and strongest when considering ‘any PD.’ Thus, the data indicate that both reliability and convergence would be improved by eliminating the specific PDs and focusing diagnosis simply on *any* PD, including PD-TS; that is, characterizing patients not by specific diagnostic labels but by the presence of moderate or greater impairment in personality functioning and by their individual trait profiles.

#### Application

These results have implications for clinical practice. First, our data indicate – through high coverage and convergence of prevalence across the two *DSM-5* PD models when *all* PD is included – that they identify largely the same set of PD patients. Second, the AMPD addresses two shortcomings of the Section-II diagnostic system. Specifically, it provides a way to understand comorbidity, in that individuals with multiple PDs may be expected to have more elevated and complex trait profiles. Conversely, it addresses within-diagnosis heterogeneity by focusing on individuals' trait profiles rather than specific criteria. That is, in the Section-II PD model, due to its polythetic diagnostic system, having diverse combinations of specific criteria (i.e. heterogeneity) is inherent in the model, yet problematic because consideration of differences among criterion combinations is not part of the model. In contrast, in the AMPD, within-PD heterogeneity results from the small number (2 to 7) of traits used to diagnose any particular PD relative to all 25. Patients could share the traits of a specific PD but differ on the other 18 to 23. However, it is more likely that they will have additional trait elevations beyond those of a specific PD because ‘comorbidity’ is roughly equally common in both models. However, this is not so problematic for the AMPD because all individuals have their own trait profile that comprehensively describes the nature of their PD characteristics. Specifically, the AMPD provides PD-TS to diagnose all PD (i.e. as well as those with one or more specific PDs) by describing all trait expressions of personality pathology using Criterion B trait. In contrast, the Section-II PD model has no systematic way of characterizing the almost 40% of individuals who meet its General PD Criterion, but who can only be diagnosed with PD-NEC/PD-NOS.

Further, the AMPD advances PD *theoretically* by distinguishing function from form; specifically, by separating personality dysfunction (Criterion A) – the core aspect of PD that is shared across all personality pathology – from the form that personality dysfunction takes, the way it is expressed via personality traits (Criterion B). That said, although this theoretical distinction has some empirical support (e.g. Hopwood et al., 2011; Sharp et al., 2015), most current measures of Criterion A and B overlap considerably. Moreover, results are mixed regarding whether Criterion A has incremental predictive power over Criterion B, whereas Criterion B almost always shows incremental predictive power over Criterion A; plus, Criterion A correlates strongly with clinical syndromes as well as PDs, indicating that current measures of the construct are not specific to PD (Roche & Jaweed, 2023; Sleep, Lynam, Widiger, Crowe, & Miller, 2019, 2020). Thus, taken together, these data – along with considerable other data published since 2013 – suggest that a shift to the AMPD is promising, although there remain areas for improvement, particularly in its assessment.

#### Practical considerations

In the introduction, we noted that use of a semi-structured interview may be impractical in many clinical settings. However, given the importance of accurate diagnosis, perhaps it is time for the field to determine how best to incorporate reliable and valid measures into standard clinical practice so as to provide a way for clinicians to use the AMPD to characterize all their PD patients' personality pathology, not just the subset that can be described with specific PDs (~60%) or diagnosed unhelpfully with PD-NEC (~40%). (We thank an anonymous reviewer for encouraging us to include this ‘editorial’ calling for higher standards for assessment in the field.) Some of this responsibility falls on clinicians to adopt ‘best practices.’ For example, clinicians refer patients for neuropsychological testing rather than judging the degree to which patients may be cognitive impaired based on an unstructured interview. Perhaps a similar standard should be applied to PD assessment, particularly if the AMPD were to be adopted by APA. Currently, the American Psychiatric Association's Clinical Practice Guidelines include only two assessment tools – for the care of patients with schizophrenia and for management of patients at risk for suicide – but perhaps there also should be assessment and treatment guidelines for PD traits, such as the Unified Protocol for the transdiagnostic treatment of negative affectivity (neuroticism; Sauer-Zavala et al., 2021).

In addition, test developers also have a responsibility to provide users with the means to score and interpret their measures; it apparently is insufficient to have a scoring key for the PID-5 available on the APA website. (An example of the type of support needed can be found at the following, which can be cited as Bryant, W.T. [date retrieved]. https://www.google.com/url?q=https://docs.google.com/spreadsheets/d/1407ffkDeXv00VmhDBwqnkExUUPZWN7tk/edit%23gid%3D303628129&source=gmail-imap&ust=1705954231000000&usg=AOvVaw0JuIAwtYu6YR8spwh1WZ1_). Rather, infrastructure similar to what Pearson Assessment offers for the MMPI-3 (Ben-Porath & Tellegen, 2020), including online scoring programs and training workshops, may be needed, but such infrastructure likely will not be developed unless practice guidelines create the need. Further, developing reliable and valid – as well as feasible – assessment tools for Criterion A (*v.* B) will likely be rather more challenging, given that structured trait assessment has a very long history, dating back to the early 1900s, whereas Criterion A lacks such a history.

Another consideration is the order of assessment. Assuming adequate infrastructure, it may be most efficient to assess Criterion B first and then, if warranted by an elevated trait profile, to assess Criterion A (plus assess Criterion B further if there is concern about the validity of self-report). (We credit and are grateful to Joshua Miller for this suggestion.) Alternatively, assessing Criterion A first would have the benefit of aligning with the *ICD-11* PD model, which parallels AMPD's Criterion A, describing three severity levels of personality impairment (mild, moderate, and severe) and provides an optional trait system for further characterization that overlaps considerably with the AMPD's.

A further practical consideration is that there currently exists only one interview designed specifically to assess the AMPD – *Structured Clinical Interview for the AMPDs* (SCID-5-AMPD; First, Skodol, Bender, & Oldham, 2018). Its three modules are extensive plus, being proprietary it is not free, which may limit its use, even in research. In brief, development of other reliable, valid, and freely available ways to assess the AMPD should be a priority for the field.

## Limitations

A primary limitation of our study is that in almost all cases the same interviewer completed the ratings for both the Section-II PD criteria and the AMPD ratings of personality dysfunction (Criterion A) and traits (Criterion B). This likely led to greater convergence between the models than if a second person had (a) made the AMPD ratings (e.g. see Few et al., 2013), (b) re-administered the interview, (c) used a different Section-II interview (e.g. Clark, Livesley, & Morey, 1997, reported a median kappa of 0.33 for ‘any PD’ between Section-II interviews), or (d) used an interview specifically targeted to the AMPD (e.g. the SCID-5-AMPD, which had not yet been developed when we began data collection in 2012). These four alternative methods would provide increasingly stringent tests of the between-model overlap.

A second limitation is that the data were collected using a particular clinical and community sample, recruited in two different ways and reflecting the demographics of the part of the U.S. where the data were collected, the particular set of clinical disorders of the sample, and the nature of recruitment. Additional samples, including from non-‘WEIRD’ (Western, Educated, Industrialized, Rich, and Demographic.) countries, are needed to test the findings' generalizability.

In brief, use of different measures of the Section-II and III PDs and of different assessors could yield different results from ours and, importantly, how those results (and ours) would converge with use in clinical practice is unknown. Nonetheless, our results provide preliminary evidence that use of the AMPD can identify largely the same overall population of patients with PD as the current Section-II set of diagnoses. They also suggest that the current set of traits used to diagnose the AMPD's specific disorders may not converge well with those of Section II and perhaps should be abandoned, given that the PD-TS diagnosis covers all of PD.

### Clinical utility

Research on the clinical utility of the AMPD (e.g., Bornstein & Natoli's, 2019 meta-analysis) indicates that clinicians who are asked to use the two systems to characterize either patients described in vignettes or chosen from their practice, and then rate the two systems on various parameters, prefer the AMPD over the Section-II model in terms of comprehensiveness in describing patients' personality problems, and usefulness in communicating with patients, formulating a therapeutic intervention, and describing patients' global personality. Further, the two models are rated equally on ease of use, and usefulness in communicating with other mental health professionals and treatment planning. The authors thus concluded that their meta-analytic findings were ‘robust and reliable’ (p. 426).

## Conclusion

We hope that – taken together – our findings and those of many other publications investigating the AMPD, the advent of the highly similar *ICD-11* model, and the aggregated clinical-utility findings will encourage greater use of the AMPD not only in research, but also in clinical settings that will lead eventually to its formal adoption by the American Psychiatric Association as its primary model for comprehensive diagnosis of all personality pathology.

## Supporting information

Clark et al. supplementary material 1 Clark et al. supplementary material

Clark et al. supplementary material 2 Clark et al. supplementary material

## Supplementary material

The supplementary material for this article can be found at https://doi.org/10.1017/S0033291724000357.
