# Supplementary material for: Personality disorder coverage, prevalence, and convergence: do the DSM-5's two models of personality disorder identify the same patients?
Source: Psychol Med. 2024 Mar 19;54(9):2210–21. doi: 10.1017/S0033291724000357 (PMC11413344; doi:10.1017/S0033291724000357)
Supplement: Clark et al. supplementary material 1 — Clark et al. supplementary material [file S0033291724000357sup001.docx]

Supplemental Table S1

*Correlations Between Section-II Personality Disorder Criterion Counts and Section-III (AMPD) Personality Disorder Trait Counts*

Section-II Personality Disorder Criterion Counts

AMPD Trait Counts PAR SZD STY ANT BOR HIS NAR AVD DND OCPD

Paranoid^a^ **.67*†** .28 **.63** .34 **.52** .20 .40 .18 .12 .21

Schizoid^a^ .33 **.69*†** .42 .05 .20 -.04 .11 .40 .09 .09

Schizotypal **.56** **.53** **.72*†** .19 .37 .09 .30 .29 .11 .18

Antisocial .35 .09 .30 **.83*†** **.57** .36 **.55** .00 .11 .15

Borderline .43 .21 .40 .46 **.73*†** .33 .33 .41 .46 .26

Histrionic^a^ .30 .05 .23 .27 **.55** **.57*†** .36 .13 .25 .22

Narcissistic .29 .01 .22 .36 .27 **.52** **.74*†** -.13 .02 .24

Avoidant .38 **.58*** .41 .03 .24 .01 .12 **.55†** .18 .18

Dependent^a^ .19 .11 .18 .06 .33 .18 .06 .41 **.76*†** .13

Obsessive-Compulsive .26 .41 .27 .02 .18 .07 .23 .25 .14 **.53*†**

*Note.* *N* = 607. Both the Section-II PDs and the AMPD traits were rated based on the Structured Interview for *DSM* Personality (SIDP; Pfohl, Blum, & Zimmerman, 1997). PAR = Paranoid PD. SZD = Schizoid PD. STY = Schizotypal PD. ANT = Antisocial PD. BOR = Borderline PD. HIS = Histrionic PD. NAR = Narcissistic PD. AVD = Avoidant PD. DND = Dependent PD. OCPD = Obsessive-Compulsive PD. Values < .19 are greyed; from .35 to .49 are underlined; from **.50** to **.59** are **bolded**; > **.60** are **bolded** and **underlined**.

* = Highest correlation in each row. †Highest correlation in each column.

^a^PDs not specified in the AMPD. These were scored using trait facets required to be in the pathological range—2‑3 of a 0-3 rating scale—identified tentatively by the *DSM-5* Personality and Personality Disorder Work Group, as follows. Paranoid PD: two or more of suspiciousness, unusual beliefs and experiences, and hostility; Schizoid PD: three or more of withdrawal, intimacy avoidance, anhedonia, and restricted affectivity; Histrionic PD: emotional lability and attention seeking; Dependent PD: separation insecurity and submissiveness. As with the primary six AM-PDs, the selected traits were required to be in the pathological range—rated 2 or 3 on a 0–3 scale. However, because the trait selections for these PDs were never validated, these results are preliminary results with these variables must be considered with caution.

Supplemental Table S2

*Base Rates and Severity (Number of Criteria Met) of 13 Common Clinical Disorders in the Overall Sample and Each Subsample*

Base Rates^a^ Severity of Those with Diagnoses (# of Criteria Met)

High-PD-Risk *p*-value of *M(SD)* *p*-value of

Diagnoses Overall Patients Community difference Overall Patients HR Community difference

Major depressive disorder 29.8 34.6 24.8 < .01 6.82 (1.26) 7.01 (1.32) 6.55 (1.12) .02

Dysthymic disorder 18.8 22.2 15.2 < .03 4.93 (1.00) 5.01 (1.03) 4.80 (0.96) < .001

Manic episode (lifetime) 17.6 25.5 9.60 < .0001 5.60 (1.18) 5.76 (1.13) 5.17 (1.22) .03

Panic disorder 23.5 31.1 15.9 < .0001 9.36 (2.14) 9.69 (2.02) 8.71 (2.24) < .02

Agoraphobia 8.88 10.5 7.28 ns 5.00 (0.00) 5.00 (0.00) 5.00 (0.00) NA

Social anxiety disorder 17.9 20.9 14.9 ns 5.00 (0.00) 5.00 (0.00) 5.00 (0.00) NA

Generalized anxiety disorder 27.5 30.7 24.2 ns 9.47 (1.69) 9.37 (1.78) 9.59 (1.59) *ns*

Obsessive-compulsive disorder 9.87 11.4 8.28 ns 3.37 (0.88) 3.31 (0.76) 3.44 (1.04) *ns*

Post-traumatic stress disorder 8.88 13.1 4.64 < .0002 15.9 (2.26) 15.8 (2.36) 16.0 (2.25) *ns*

Bulimia nervosa 1.15 0.98 1.15 ns 4.00 (0.00) 4.00 (0.00) 4.00 (0.00) NA

Binge eating disorder 3.29 4.25 2.32 ns 4.00 (0.79) 4.15 (0.80) 3.71 (0.76) *ns*

Alcohol use disorder 18.4 19.3 17.6 ns 5.29 (3.06) 6.64 (3.07) 3.77 (0.97) < .001

Substance use disorder 14.0 18.6 9.27 <.0008 5.69 (3.04 6.39 (3.21) 4.29 (2.09) < .001

*Note. N* = 608; patient *n* = 306; high-PD-risk community *n* = 301. ^a^Current unless otherwise specified.

Supplemental Table S3

*Base Rates of Section II and AMPD Personality Disorders in the Overall Sample*

*and Each Subsample*

Base Rates

High-PD-Risk *p*-value of

Diagnoses Overall Patients Community difference

Section-II PDs

Antisocial 8.24 12.13 4.30 <.0004

Borderline 9.72 15.08 4.30 <.0001

Avoidant 11.7 13.11 10.3 *ns*

Narcissistic 4.94 7.21 2.65 <.01

Obsessive-Compulsive 8.07 8.2 7.95 *ns*

Schizotypal 2.80 4.26 1.32 <.03

Paranoid 6.26 9.18 3.31 <.03

Schizoid 1.98 2.95 0.99 *ns*

Histrionic 1.32 2.3 0.33 <.04

Dependent 3.13 3.61 2.65 *ns*

AMPD

Antisocial 2.80 3.93 1.66 *ns*

Borderline 13.8 20.0 7.62 <.0001

Avoidant 6.26 9.84 2.65 <.0003

Narcissistic 5.43 7.54 3.31 < .03

Obsessive-Compulsive 2.97 4.26 1.66 *ns*

Schizotypal 5.93 9.84 1.99 <.0001

Paranoid^a^ 19.1 25.9 12.3 <.0001

Schizoid^a^ 6.75 8.52 4.97 *ns*

Histrionic^a^ 4.78 6.23 3.31 *ns*

Dependent^a^ 5.27 6.23 4.30 *ns*

*Note*. Overall *N* = 607. Patient *n* = 305. High-PD-risk community *n* = 302.

^a^Personality disorders (PDs) not specified in the Alternative *DSM-5* Model of PD. These PDs were scored using trait facets identified tentatively by the *DSM-5* Personality and Personality Disorder Work Group as follows: Paranoid PD—two or more of suspiciousness, unusual beliefs and experiences, and hostility; Schizoid PD—three or more of withdrawal, intimacy avoidance, anhedonia, and restricted affectivity; Histrionic PD—emotional lability and attention seeking; Dependent PD—separation insecurity and submissiveness. As with the primary six AM-PDs, the selected traits were required to be in the pathological range—2‑3 of a 0-3 rating scale. However, because the trait selections for these PDs were never validated, these results are preliminary and results with these variables must be considered with caution.

Supplemental Table S4a

*Percentage of Comorbid Diagnoses Between Each Pair of Section II Personality Disorders in the Patient Subsample*

Diagnosis

Diagnosis PAR SZD STP ANT BOR HIS NAR AVD DPN OC Total

Paranoid PD 14.3 25.0 28.6 **46.4*†** 7.1 28.6 21.4 3.6 17.9 28

Schizoid PD **44.4*** **33.3†** 22.2 22.2 0.0 0.0 **44.4*** 0.0 11.1 9

Schizotypal PD **53.9*†** 23.1† **30.8** **46.2** 0.0 **38.5** 23.1 0.0 23.1 13

Antisocial PD 21.6 5.4 10.8 **32.4*** 5.4 27.0 18.9 2.7 16.2 37

Borderline PD 28.3* 4.4 13.0 26.1 6.5 19.6 28.3* 6.5 21.7 46

Histrionic PD 28.6 0.0 0.0 28.6 **42.9** **57.1*†** 0.0 0.0 0.0 7

Narcissistic PD **36.4** 0.0 22.7 **45.5*†** **40.9** 18.2† 4.6 0.0 27.3† 22

Avoidant PD 15.0 10.0 7.5 17.5 **32.5*** 0.0 2.5 17.5† 12.5 40

Dependent PD 9.1 0.0 0.0 9.1 27.3 0.0 0.0 **63.6*†** 9.1 11

Obssv-Cmplsv PD 20.0 4.0 12.0 24.0 **40.0*** 4.0 24.0 20.0 4.0 25

*Note*. Numbers indicate the percentage of the personality disorder (PD) in that row that is comorbid with the diagnosis in each column. For example, of the 22 individuals diagnosed with Narcissistic PD, 40.9%were also diagnosed with Borderline PD, whereas of the 46 individuals diagnosed with Borderline PD, 19.6% were also diagnosed with Narcissistic PD.

PAR = Paranoid. SZD= Schizoid. STP = Schizotypal. ANT = Antisocial. BOR = Borderline. HIS = Histrionic. NAR = Narcissistic. AVD = Avoidant. DEP = Dependent. OC = Obsessive Compulsive. Obssv-Cmplsv = Obsessive Compulsive.

Percentages > 30% are in **bold**; those between 20% and 29% are underlined.

* = Highest percentage of comorbidity in each row. †Highest percentage of comorbidity in each column. Thus, *† indicates pairs of PD diagnoses that tend to be specifically comorbid with each other.

Supplemental Table S4b

*Percentage of Comorbid Diagnoses Between Each Pair of Section II Personality Disorders in the High-PD-Risk Community Subsample*

Diagnosis

Diagnosis PAR SZD STP ANT BOR HIS NAR AVD DPN OC Total

Paranoid PD 10.0 10.0† 20.0 20.0 0.0 20.0 **30.0*** 0.0 10.0 10

Schizoid PD **33.3*†** 0.0 **33.3*†** 0.0 0.0 **33.3*†** 0.0 0.0 0.0 3

Schizotypal PD 25.0* 0.0 0.0 0.0 0.0 0.0 0.0 0.0 0.0 4

Antisocial PD 15.4 7.7 0.0 23.1* 0.0 15.4 15.4 7.7 23.1* 13

Borderline PD 15.4 0.0 0.0 23.1 0.0 15.4 **53.9*†** 7.7 30.8† 13

Histrionic PD 0.0 0.0 0.0 0.0 0.0 100.0*† 0.0 0.0 0.0 1

Narcissistic PD 25.0* 15.5† 0.0 25.0* 25.0*† 12.5 15.5 0.0 25.0* 8

Avoidant PD 9.7 0.0 0.0 6.5 22.6 0.0 3.2 12.9† 25.8* 31

Dependent PD 0.0 0.0 0.0 12.5 12.5 0.0 0.0 **50.0*** 12.5 8

Obssv-Cmplsv PD 4.2 0.0 0.0 12.5 16.7 0.0 8.3 33.3* 4.2 24

*Note*. Numbers indicate the percentage of the personality disorder (PD) in that row that is comorbid with the diagnosis in each column. For example, of the 31 individuals diagnosed with Avoidant PD, 22.6% were also diagnosed with Borderline PD, whereas of the 13 individuals diagnosed with Borderline PD, 53.9% were also diagnosed with Avoidant PD.

PAR = Paranoid. SZD= Schizoid. STP = Schizotypal. ANT = Antisocial. BOR = Borderline. HIS = Histrionic. NAR = Narcissistic. AVD = Avoidant. DEP = Dependent. OC = Obsessive Compulsive. Obssv-Cmplsv = Obsessive Compulsive.

Percentages > 30% are in **bold**; those between 20% and 29% are underlined.

* = Highest percentage of comorbidity in each row. †Highest percentage of comorbidity in each column. Thus, *† indicates pairs of PD diagnoses that tend to be specifically comorbid with each other.

Supplemental Table S4c

*Percentage of Comorbid Diagnoses Between Each Pair of Section-III Personality Disorders in the Patient Subsample*

PD Diagnosis

Diagnosis ANT AVD BOR NAR OC STP PAR SZD HIS DPN Total

Antisocial PD 0.0 **50.0†** **41.7†** 0.0 8.3 **66.7** 8.3 25.0 0.0 12

Avoidant PD 0.0 **43.3*** 10.0 20.0† **40.0†** **70.0** **56.7** 3.3 23.3 30

Borderline PD 9.8 21.3 16.4 6.6 23.0* **72.1** 18.0 21.3 16.4 61

Narcissistic PD 21.7† 13.0 **43.5*** 13.0 17.4 **69.6** 13.0 **56.5** 4.4 23

Obssv-Cmplsv PD 0.0 **46.2*†** **30.8** 23.1 **38.5** **53.9** **53.9** 15.4 15.4 13

Schizotypal PD 3.3 **40.0** **46.7*** 13.3 16.7 **93.3** **33.3** 13.3 **33.3** 30

Paranoid PD^a^ 10.1 26.6 **55.7** 20.3 8.9 **35.4** 24.1 13.9 11.4 79

Schizoid PD^a^ 3.9 **65.4** **42.3** 11.5 26.9 **38.5** **73.1** 7.7 11.5 26

Histrionic PD^a^ 15.8 5.3 **68.4** **68.4** 10.5 21.1 **57.9** 10.5 5.3 19

Dependent PD^a^ 0.0 **36.8** **52.6** 5.3 10.5 5.3 **47.4** 15.8 5.3 19

*Note*. Numbers indicate the percentage of the personality disorder (PD) in that row that is comorbid with the diagnosis in each column. For example, of the 30 individuals diagnosed with Avoidant PD, 43.5% were also diagnosed with Borderline PD, whereas of the 61 individuals diagnosed with Borderline PD, 13% were also diagnosed with Avoidant PD. PAR = Paranoid. SZD= Schizoid. STP = Schizotypal. ANT = Antisocial. BOR = Borderline. HIS = Histrionic. NAR = Narcissistic. AVD = Avoidant. DEP = Dependent. OC = Obsessive Compulsive. Obssv-Cmplsv = Obsessive Compulsive. Percentages > 30% are in **bold**; those between 20% and 29% are underlined. For the six specific AMPDs whose traits have been validated: *Highest percentage of comorbidity in each row. †Highest percentage of comorbidity in each column. Thus, *† indicates pairs of PD diagnoses that are specifically comorbid with each other.

^a^Personality disorders (PDs) not specified in the Alternative *DSM-5* Model of PD. These PDs were scored using trait facets identified tentatively by the *DSM-5* Personality and Personality Disorder Work Group, as follows. Paranoid PD—two or more of suspiciousness, unusual beliefs and experiences, and hostility; Schizoid PD—three or more of withdrawal, intimacy avoidance, anhedonia, and restricted affectivity; Histrionic PD—emotional lability and attention seeking; Dependent PD—separation insecurity and submissiveness. As with the primary six AM-PDs, the selected traits were required to be in the pathological range—2‑3 of a 0-3 rating scale. However, because the trait selections for these PDs were never validated, these results are preliminary and results with these variables must be considered with caution.

Supplemental Table S4d

*Percentage of Comorbid Diagnoses Between Each Pair of Section-III Personality Disorders in the High-PD-Risk Community Subsample*

PD Diagnosis

Diagnosis ANT AVD BOR NAR OC STP PAR SZD HIS DPN Total

Antisocial PD 0.0 20.0 **40.0*†** 0.0 0.0 **80.0** 20.0 0.0 0.0 5

Avoidant PD 0.0 12.5* 12.5* 12.5*† 12.5* **37.5** **50.0** 12.5 12.5 8

Borderline PD 4.4 4.4 4.6 0.0 17.4*† **52.2** 8.7 17.4 26.1 23

Narcissistic PD 20.0*† 10.0 10.0 0.0 10.0 **60.0** 10.0 40.0 10.0 10

Obssv-Cmplsv PD 0.0 20.0*† 0.0 0.0 0.0 **40.0** **40.0** 0.0 20.0 5

Schizotypal PD 0.0 16.7 **66.7*†** 16.7 0.0 **100.0** **33.3** 16.7 16.7 6

Paranoid PD^a^ 10.8 8.1 **32.4** 16.2 5.4 16.2 16.2 10.814 10.1 37

Schizoid PD^a^ 6.7 26.7 13.3 6.7 13.3 13.3 **40.0** 0.0 6.7 15

Histrionic PD^a^ 0.0 10.0 **40.0** **40.0** 0.0 10.0 **40.0** 0.0 **30.0** 10

Dependent PD^a^ 0.0 7.7 **46.2** 7.7 7.7 7.7 **30.8** 7.7 23.1 13

*Note*. Numbers indicate the percentage of the personality disorder (PD) in that row that is comorbid with the diagnosis in each column. For example, of the 23 individuals diagnosed with Borderline PD, 17.4% were also diagnosed with Schizotypal PD, whereas of the 6 individuals diagnosed with Schizotypal PD, 66.7% were also diagnosed with Borderline PD. PAR = Paranoid. SZD= Schizoid. STP = Schizotypal. ANT = Antisocial. BOR = Borderline. HIS = Histrionic. NAR = Narcissistic. AVD = Avoidant. DEP = Dependent. OC = Obsessive Compulsive. Obssv-Cmplsv = Obsessive Compulsive. Percentages > 30% are in **bold**; those between 20% and 29% are underlined. For the six specific AMPDs who traits have been validated, * = Highest percentage of comorbidity in each row. †Highest percentage of comorbidity in each column. Thus, *† indicates pairs of PD diagnoses that tend to be specifically comorbid with each other.

^a^Personality disorders (PDs) not specified in the Alternative *DSM-5* Model of PD. These PDs were scored using trait facets identified tentatively by the *DSM-5* Personality and Personality Disorder Work Group, as follows. Paranoid PD—two or more of suspiciousness, unusual beliefs and experiences, and hostility; Schizoid PD—three or more of withdrawal, intimacy avoidance, anhedonia, and restricted affectivity; Histrionic PD—emotional lability and attention seeking; Dependent PD—separation insecurity and submissiveness. As with the primary six AM-PDs, the selected traits were required to be in the pathological range—2‑3 of a 0-3 rating scale. However, because the trait selections for these PDs were never validated, these results are preliminary and results with these variables must be considered with caution.

Supplemental Table S5

*Descriptive Statistics:* *Structured Interview for* DSM-5 *Personality (SIDP) AMPD ratings*

Cronbach’s α McDonald’s

Variable Mean *SD* Range / AIC ω

Criterion A

Overall, rated 0.50 0.50 0 – 1 .--^a^ .--

Identity 1.35 1.01 0 – 4 .-- .--

Self-direction 1.20 0.90 0 – 4 .-- .--

Empathy 0.98 0.93 0 – 4 .-- .--

Intimacy 1.34 1.04 0 – 4 .-- .--

Subcomponents sum 4.87 3.24 0 – 15 .85 / .59 .85

Overall, scored 0.39 0.49 0 – 1

Criterion B

FACETS

Emotional Lability 0.97 0.90 0 – 3 .-- .--

Anxiousness 0.93 0.89 0 – 3 .-- .--

Separation Insecurity 0.68 0.84 0 – 3 .-- .--

Submissiveness 0.75 0.74 0 – 3 .-- .--

Hostility 1.01 0.83 0 – 3 .-- .--

Perseveration 0.43 0.70 0 – 3 .-- .--

Depressivity 1.00 0.90 0 – 3 .-- .--

Withdrawal 0.97 0.93 0 – 3 .-- .--

Intimacy Avoidance 0.65 0.82 0 – 3 .-- .--

Anhedonia 0.51 0.75 0 – 3 .-- .--

Restricted Affectivity 0.38 0.66 0 – 3 .-- .--

Suspiciousness 1.02 0.92 0 – 3 .-- .--

Manipulativeness 0.48 0.79 0 – 3 .-- .--

Deceitfulness 0.49 0.75 0 – 3 .-- .--

Grandiosity 0.64 0.81 0 – 3 .-- .--

Attention seeking 0.49 0.71 0 – 3 .-- .--

Callousness 0.51 0.70 0 – 3 .-- .--

Irresponsibility 0.86 0.89 0 – 3 .-- .--

Impulsivity 0.75 0.87 0 – 3 .-- .--

Distractibility 0.96 0.95 0 – 3 .-- .--

Risk Taking 0.47 0.73 0 – 3 .-- .--

Rigid Perfectionism 0.34 0.68 0 – 3 .-- .--

Unus. Beliefs/ Expers. 0.62 0.78 0 – 3 .-- .--

Eccentricity 0.28 0.62 0 – 3 .-- .--

Cog./ Percept. Dysfunc. 0.56 0.76 0 – 3 .-- .--

*(table continues)*

Supplemental Table S5 (cont.)

Cronbach’s α McDonald’s

Variable Mean *SD* Range / AIC ω

DOMAINS CALCULATED FROM “BEST 3” FACETS

Negative Affectivity 0.86 0.68 0–2.67 .68 / .41 .68

Detachment 0.71 0.68 0–2.67 .74 / .49 .76

Antagonism 0.54 0.65 0 – 3 .77 / .53 .79

Disinhibition 0.73 0.67 0–2.67 .68 / .41 .74

Psychoticism 0.49 0.59 0 – 3 .75 / .50 .76

*Note.* *N*=607. *α* = alpha. *ω* = omega. *SD* = Standard deviation. Unus. Beliefs/ Expers. = Unusual Beliefs and Experiences. Cog./ Percept. Dysfunc. = Cognitive and Perceptual Dysfunction. AIC = average interitem correlation.

^a^Not applicable
