# Supplementary material for: Personality disorder coverage, prevalence, and convergence: do the DSM-5's two models of personality disorder identify the same patients?
Source: Psychol Med. 2024 Mar 19;54(9):2210–21. doi: 10.1017/S0033291724000357 (PMC11413344; doi:10.1017/S0033291724000357)
Supplement: Clark et al. supplementary material 2 — Clark et al. supplementary material [file S0033291724000357sup002.pdf]

Supplemental Table S1

Correlations Between Section-II Personality Disorder Criterion Counts and Section-III (AMPD) Personality Disorder Trait Counts

	Section-II Personality Disorder Criterion Counts									
AMPD Trait Counts	PAR	SZD	STY	ANT	BOR	HIS	NAR	AVD	DND	OCPD
Paranoid[a]	**.67*†**	.28	**.63**	.34	**.52**	.20	.40	.18	.12	.21
Schizoid[a]	.33	**.69*†**	.42	.05	.20	-.04	.11	.40	.09	.09
Schizotypal	**.56**	**.53**	**.72*†**	.19	.37	.09	.30	.29	.11	.18
Antisocial	.35	.09	.30	**.83*†**	**.57**	.36	**.55**	.00	.11	.15
Borderline	.43	.21	.40	.46	**.73*†**	.33	.33	.41	.46	.26
Histrionic[a]	.30	.05	.23	.27	**.55**	**.57*†**	.36	.13	.25	.22
Narcissistic	.29	.01	.22	.36	.27	**.52**	**.74*†**	-.13	.02	.24
Avoidant	.38	**.58***	.41	.03	.24	.01	.12	**.55†**	.18	.18
Dependent[a]	.19	.11	.18	.06	.33	.18	.06	.41	**.76*†**	.13
Obsessive-Compulsive	.26	.41	.27	.02	.18	.07	.23	.25	.14	**.53*†**

Note. N = 607. Both the Section-II PDs and the AMPD traits were rated based on the Structured Interview for *DSM* Personality (SIDP; Pfohl, Blum, & Zimmerman, 1997). PAR = Paranoid PD. SZD = Schizoid PD. STY = Schizotypal PD. ANT = Antisocial PD. BOR = Borderline PD. HIS = Histrionic PD. NAR = Narcissistic PD. AVD = Avoidant PD. DND = Dependent PD. OCPD = Obsessive-Compulsive PD. Values ≤ .19 are greyed; from .35 to .49 are underlined; from **.50** to **.59** are **bolded**; ≥ **.60** are **bolded** and underlined.

* = Highest correlation in each row. †Highest correlation in each column.

[a]PDs not specified in the AMPD. These were scored using trait facets required to be in the pathological range—2-3 of a 0-3 rating scale—identified tentatively by the *DSM-5* Personality and Personality Disorder Work Group, as follows. Paranoid PD: two or more of suspiciousness, unusual beliefs and experiences, and hostility; Schizoid PD: three or more of withdrawal, intimacy avoidance, anhedonia, and restricted affectivity; Histrionic PD: emotional lability and attention seeking; Dependent PD: separation insecurity and submissiveness. As with the primary six AM-PDs, the selected traits were required to be in the pathological range—rated 2 or 3 on a 0–3 scale. However, because the trait selections for these PDs were never validated, these results are preliminary results with these variables must be considered with caution.

Supplemental Table S2

Base Rates and Severity (Number of Criteria Met) of 13 Common Clinical Disorders in the Overall Sample and Each Subsample

	Base Rates[a]				Severity of Those with Diagnoses (# of Criteria Met)			
					M(SD)			
Diagnoses	Overall	Patients	High-PD-Risk Community	*p*-value of difference	Overall	Patients	HR Community	*p*-value of difference
Major depressive disorder	29.8	34.6	24.8	< .01	6.82 (1.26)	7.01 (1.32)	6.55 (1.12)	.02
Dysthymic disorder	18.8	22.2	15.2	< .03	4.93 (1.00)	5.01 (1.03)	4.80 (0.96)	< .001
Manic episode (lifetime)	17.6	25.5	9.60	< .0001	5.60 (1.18)	5.76 (1.13)	5.17 (1.22)	.03
Panic disorder	23.5	31.1	15.9	< .0001	9.36 (2.14)	9.69 (2.02)	8.71 (2.24)	< .02
Agoraphobia	8.88	10.5	7.28	ns	5.00 (0.00)	5.00 (0.00)	5.00 (0.00)	NA
Social anxiety disorder	17.9	20.9	14.9	ns	5.00 (0.00)	5.00 (0.00)	5.00 (0.00)	NA
Generalized anxiety disorder	27.5	30.7	24.2	ns	9.47 (1.69)	9.37 (1.78)	9.59 (1.59)	*ns*
Obsessive-compulsive disorder	9.87	11.4	8.28	ns	3.37 (0.88)	3.31 (0.76)	3.44 (1.04)	*ns*
Post-traumatic stress disorder	8.88	13.1	4.64	< .0002	15.9 (2.26)	15.8 (2.36)	16.0 (2.25)	*ns*
Bulimia nervosa	1.15	0.98	1.15	ns	4.00 (0.00)	4.00 (0.00)	4.00 (0.00)	NA
Binge eating disorder	3.29	4.25	2.32	ns	4.00 (0.79)	4.15 (0.80)	3.71 (0.76)	*ns*
Alcohol use disorder	18.4	19.3	17.6	ns	5.29 (3.06)	6.64 (3.07)	3.77 (0.97)	< .001
Substance use disorder	14.0	18.6	9.27	<.0008	5.69 (3.04	6.39 (3.21)	4.29 (2.09)	< .001

Note. N = 608; patient n = 306; high-PD-risk community n = 301. [a]Current unless otherwise specified.

Supplemental Table S3

Base Rates of Section II and AMPD Personality Disorders in the Overall Sample and Each Subsample

	Base Rates			
Diagnoses	Overall	Patients	High-PD-Risk Community	*p*-value of difference
		Section-II PDs		
Antisocial	8.24	12.13	4.30	<.0004
Borderline	9.72	15.08	4.30	<.0001
Avoidant	11.7	13.11	10.3	*ns*
Narcissistic	4.94	7.21	2.65	<.01
Obsessive-Compulsive	8.07	8.2	7.95	*ns*
Schizotypal	2.80	4.26	1.32	<.03
Paranoid	6.26	9.18	3.31	<.03
Schizoid	1.98	2.95	0.99	*ns*
Histrionic	1.32	2.3	0.33	<.04
Dependent	3.13	3.61	2.65	*ns*
		AMPD		
Antisocial	2.80	3.93	1.66	*ns*
Borderline	13.8	20.0	7.62	<.0001
Avoidant	6.26	9.84	2.65	<.0003
Narcissistic	5.43	7.54	3.31	< .03
Obsessive-Compulsive	2.97	4.26	1.66	*ns*
Schizotypal	5.93	9.84	1.99	<.0001
Paranoid[a]	19.1	25.9	12.3	<.0001
Schizoid[a]	6.75	8.52	4.97	*ns*
Histrionic[a]	4.78	6.23	3.31	*ns*
Dependent[a]	5.27	6.23	4.30	*ns*

Note. Overall *N* = 607. Patient *n* = 305. High-PD-risk community *n* = 302.
[a]Personality disorders (PDs) not specified in the Alternative *DSM-5* Model of PD. These PDs were scored using trait facets identified tentatively by the *DSM-5* Personality and Personality Disorder Work Group as follows: Paranoid PD—two or more of suspiciousness, unusual beliefs and experiences, and hostility; Schizoid PD—three or more of withdrawal, intimacy avoidance, anhedonia, and restricted affectivity; Histrionic PD—emotional lability and attention seeking; Dependent PD—separation insecurity and submissiveness. As with the primary six AM-PDs, the selected traits were required to be in the pathological range—2-3 of a 0-3 rating scale. However, because the trait selections for these PDs were never validated, these results are preliminary and results with these variables must be considered with caution.

Supplemental Table S4a

Percentage of Comorbid Diagnoses Between Each Pair of Section II Personality Disorders in the Patient Subsample

	Diagnosis										
Diagnosis	PAR	SZD	STP	ANT	BOR	HIS	NAR	AVD	DPN	OC	Total
Paranoid PD		14.3	25.0	28.6	**46.4*†**	7.1	28.6	21.4	3.6	17.9	28
Schizoid PD	**44.4***		**33.3†**	22.2	22.2	0.0	0.0	**44.4***	0.0	11.1	9
Schizotypal PD	**53.9*†**	23.1†		**30.8**	**46.2**	0.0	**38.5**	23.1	0.0	23.1	13
Antisocial PD	21.6	5.4	10.8		**32.4***	5.4	27.0	18.9	2.7	16.2	37
Borderline PD	28.3*	4.4	13.0	26.1		6.5	19.6	28.3*	6.5	21.7	46
Histrionic PD	28.6	0.0	0.0	28.6	**42.9**		**57.1*†**	0.0	0.0	0.0	7
Narcissistic PD	**36.4**	0.0	22.7	**45.5*†**	**40.9**	18.2†		4.6	0.0	27.3†	22
Avoidant PD	15.0	10.0	7.5	17.5	**32.5***	0.0	2.5		17.5†	12.5	40
Dependent PD	9.1	0.0	0.0	9.1	27.3	0.0	0.0	**63.6*†**		9.1	11
Obssv-Cmplsv PD	20.0	4.0	12.0	24.0	**40.0***	4.0	24.0	20.0	4.0		25

Note. Numbers indicate the percentage of the personality disorder (PD) in that row that is comorbid with the diagnosis in each column. For example, of the 22 individuals diagnosed with Narcissistic PD, 40.9%were also diagnosed with Borderline PD, whereas of the 46 individuals diagnosed with Borderline PD, 19.6% were also diagnosed with Narcissistic PD.

PAR = Paranoid. SZD= Schizoid. STP = Schizotypal. ANT = Antisocial. BOR = Borderline. HIS = Histrionic. NAR = Narcissistic. AVD = Avoidant. DEP = Dependent. OC = Obsessive Compulsive. Obssv-Cmplsv = Obsessive Compulsive.

Percentages $\geq$ 30% are in **bold**; those between 20% and 29% are underlined.

* = Highest percentage of comorbidity in each row. †Highest percentage of comorbidity in each column. Thus, *† indicates pairs of PD diagnoses that tend to be specifically comorbid with each other.

Supplemental Table S4b

Percentage of Comorbid Diagnoses Between Each Pair of Section II Personality Disorders in the High-PD-Risk Community Subsample

	Diagnosis										
Diagnosis	PAR	SZD	STP	ANT	BOR	HIS	NAR	AVD	DPN	OC	Total
Paranoid PD		10.0	10.0†	20.0	20.0	0.0	20.0	**30.0***	0.0	10.0	10
Schizoid PD	**33.3*†**		0.0	**33.3*†**	0.0	0.0	**33.3*†**	0.0	0.0	0.0	3
Schizotypal PD	25.0*	0.0		0.0	0.0	0.0	0.0	0.0	0.0	0.0	4
Antisocial PD	15.4	7.7	0.0		23.1*	0.0	15.4	15.4	7.7	23.1*	13
Borderline PD	15.4	0.0	0.0	23.1		0.0	15.4	**53.9*†**	7.7	30.8†	13
Histrionic PD	0.0	0.0	0.0	0.0	0.0	100.0*†		0.0	0.0	0.0	1
Narcissistic PD	25.0*	15.5†	0.0	25.0*	25.0*†	12.5		15.5	0.0	25.0*	8
Avoidant PD	9.7	0.0	0.0	6.5	22.6	0.0	3.2		12.9†	25.8*	31
Dependent PD	0.0	0.0	0.0	12.5	12.5	0.0	0.0	**50.0***		12.5	8
Obssv-Cmplsv PD	4.2	0.0	0.0	12.5	16.7	0.0	8.3	33.3*	4.2		24

Note. Numbers indicate the percentage of the personality disorder (PD) in that row that is comorbid with the diagnosis in each column. For example, of the 31 individuals diagnosed with Avoidant PD, 22.6% were also diagnosed with Borderline PD, whereas of the 13 individuals diagnosed with Borderline PD, 53.9% were also diagnosed with Avoidant PD.

PAR = Paranoid. SZD= Schizoid. STP = Schizotypal. ANT = Antisocial. BOR = Borderline. HIS = Histrionic. NAR = Narcissistic. AVD = Avoidant. DEP = Dependent. OC = Obsessive Compulsive. Obssv-Cmplsv = Obsessive Compulsive.

Percentages ≥ 30% are in **bold**; those between 20% and 29% are underlined.

* = Highest percentage of comorbidity in each row. †Highest percentage of comorbidity in each column. Thus, *† indicates pairs of PD diagnoses that tend to be specifically comorbid with each other.

Supplemental Table S4c

Percentage of Comorbid Diagnoses Between Each Pair of Section-III Personality Disorders in the Patient Subsample

	PD Diagnosis										
Diagnosis	ANT	AVD	BOR	NAR	OC	STP	PAR	SZD	HIS	DPN	Total
Antisocial PD		0.0	**50.0†**	**41.7†**	0.0	8.3	**66.7**	8.3	25.0	0.0	12
Avoidant PD	0.0		**43.3***	10.0	20.0†	**40.0†**	**70.0**	**56.7**	3.3	23.3	30
Borderline PD	9.8	21.3		16.4	6.6	23.0*	**72.1**	18.0	21.3	16.4	61
Narcissistic PD	21.7†	13.0	**43.5***		13.0	17.4	**69.6**	13.0	**56.5**	4.4	23
Obssv-Cmplsv PD	0.0	**46.2*†**	**30.8**	23.1		**38.5**	**53.9**	**53.9**	15.4	15.4	13
Schizotypal PD	3.3	**40.0**	**46.7***	13.3	16.7		**93.3**	**33.3**	13.3	**33.3**	30
Paranoid PD[a]	10.1	26.6	**55.7**	20.3	8.9	**35.4**		24.1	13.9	11.4	79
Schizoid PD[a]	3.9	**65.4**	**42.3**	11.5	26.9	**38.5**	**73.1**		7.7	11.5	26
Histrionic PD[a]	15.8	5.3	**68.4**	**68.4**	10.5	21.1	**57.9**	10.5		5.3	19
Dependent PD[a]	0.0	**36.8**	**52.6**	5.3	10.5	5.3	**47.4**	15.8	5.3		19

Note. Numbers indicate the percentage of the personality disorder (PD) in that row that is comorbid with the diagnosis in each column. For example, of the 30 individuals diagnosed with Avoidant PD, 43.5% were also diagnosed with Borderline PD, whereas of the 61 individuals diagnosed with Borderline PD, 13% were also diagnosed with Avoidant PD. PAR = Paranoid. SZD= Schizoid. STP = Schizotypal. ANT = Antisocial. BOR = Borderline. HIS = Histrionic. NAR = Narcissistic. AVD = Avoidant. DEP = Dependent. OC = Obsessive Compulsive. Obssv-Cmplsv = Obsessive Compulsive. Percentages ≥ 30% are in **bold**; those between 20% and 29% are underlined. For the six specific AMPDs whose traits have been validated: *Highest percentage of comorbidity in each row. †Highest percentage of comorbidity in each column. Thus, *† indicates pairs of PD diagnoses that are specifically comorbid with each other.

[a]Personality disorders (PDs) not specified in the Alternative *DSM-5* Model of PD. These PDs were scored using trait facets identified tentatively by the *DSM-5* Personality and Personality Disorder Work Group, as follows. Paranoid PD—two or more of suspiciousness, unusual beliefs and experiences, and hostility; Schizoid PD—three or more of withdrawal, intimacy avoidance, anhedonia, and restricted affectivity; Histrionic PD—emotional lability and attention seeking; Dependent PD—separation insecurity and submissiveness. As with the primary six AM-PDs, the selected traits were required to be in the pathological range—2-3 of a 0-3 rating scale. However, because the trait selections for these PDs were never validated, these results are preliminary and results with these variables must be considered with caution.

Supplemental Table S4d

Percentage of Comorbid Diagnoses Between Each Pair of Section-III Personality Disorders in the High-PD-Risk Community Subsample

	PD Diagnosis										
Diagnosis	ANT	AVD	BOR	NAR	OC	STP	PAR	SZD	HIS	DPN	Total
Antisocial PD		0.0	20.0	**40.0*†**	0.0	0.0	**80.0**	20.0	0.0	0.0	5
Avoidant PD	0.0		12.5*	12.5*	12.5*†	12.5*	**37.5**	**50.0**	12.5	12.5	8
Borderline PD	4.4	4.4		4.6	0.0	17.4*†	**52.2**	8.7	17.4	26.1	23
Narcissistic PD	20.0*†	10.0	10.0		0.0	10.0	**60.0**	10.0	40.0	10.0	10
Obssv-Cmplsv PD	0.0	20.0*†	0.0	0.0		0.0	**40.0**	**40.0**	0.0	20.0	5
Schizotypal PD	0.0	16.7	**66.7*†**	16.7		0.0	**100.0**	**33.3**	16.7	16.7	6
Paranoid PD[a]	10.8	8.1	**32.4**	16.2	5.4	16.2		16.2	10.814	10.1	37
Schizoid PD[a]	6.7	26.7	13.3	6.7	13.3	13.3	**40.0**		0.0	6.7	15
Histrionic PD[a]	0.0	10.0	**40.0**	**40.0**	0.0	10.0	**40.0**	0.0		**30.0**	10
Dependent PD[a]	0.0	7.7	**46.2**	7.7	7.7	7.7	**30.8**	7.7	23.1		13

Note. Numbers indicate the percentage of the personality disorder (PD) in that row that is comorbid with the diagnosis in each column. For example, of the 23 individuals diagnosed with Borderline PD, 17.4% were also diagnosed with Schizotypal PD, whereas of the 6 individuals diagnosed with Schizotypal PD, 66.7% were also diagnosed with Borderline PD. PAR = Paranoid. SZD= Schizoid. STP = Schizotypal. ANT = Antisocial. BOR = Borderline. HIS = Histrionic. NAR = Narcissistic. AVD = Avoidant. DEP = Dependent. OC = Obsessive Compulsive. Obssv-Cmplsv = Obsessive Compulsive. Percentages ≥ 30% are in **bold**; those between 20% and 29% are underlined. For the six specific AMPDs who traits have been validated, * = Highest percentage of comorbidity in each row. †Highest percentage of comorbidity in each column. Thus, *† indicates pairs of PD diagnoses that tend to be specifically comorbid with each other.

[a]Personality disorders (PDs) not specified in the Alternative *DSM-5* Model of PD. These PDs were scored using trait facets identified tentatively by the *DSM-5* Personality and Personality Disorder Work Group, as follows. Paranoid PD—two or more of suspiciousness, unusual beliefs and experiences, and hostility; Schizoid PD—three or more of withdrawal, intimacy avoidance, anhedonia, and restricted affectivity; Histrionic PD—emotional lability and attention seeking; Dependent PD—separation insecurity and submissiveness. As with the primary six AM-PDs, the selected traits were required to be in the pathological range—2-3 of a 0-3 rating scale. However, because the trait selections for these PDs were never validated, these results are preliminary and results with these variables must be considered with caution.

Supplemental Table S5

Descriptive Statistics: Structured Interview for DSM-5 *Personality (SIDP) AMPD ratings*

Variable	Mean	*SD*	Range	Cronbach's α / AIC	McDonald's ω
		Criterion A			
Overall, rated	0.50	0.50	0 – 1	--[a]	--
Identity	1.35	1.01	0 – 4	--	--
Self-direction	1.20	0.90	0 – 4	--	--
Empathy	0.98	0.93	0 – 4	--	--
Intimacy	1.34	1.04	0 – 4	--	--
Subcomponents sum	4.87	3.24	0 – 15	.85 / .59	.85
Overall, scored	0.39	0.49	0 – 1		
		Criterion B			
FACETS					
Emotional Lability	0.97	0.90	0 – 3	--	--
Anxiousness	0.93	0.89	0 – 3	--	--
Separation Insecurity	0.68	0.84	0 – 3	--	--
Submissiveness	0.75	0.74	0 – 3	--	--
Hostility	1.01	0.83	0 – 3	--	--
Perseveration	0.43	0.70	0 – 3	--	--
Depressivity	1.00	0.90	0 – 3	--	--
Withdrawal	0.97	0.93	0 – 3	--	--
Intimacy Avoidance	0.65	0.82	0 – 3	--	--
Anhedonia	0.51	0.75	0 – 3	--	--
Restricted Affectivity	0.38	0.66	0 – 3	--	--
Suspiciousness	1.02	0.92	0 – 3	--	--
Manipulativeness	0.48	0.79	0 – 3	--	--
Deceitfulness	0.49	0.75	0 – 3	--	--
Grandiosity	0.64	0.81	0 – 3	--	--
Attention seeking	0.49	0.71	0 – 3	--	--
Callousness	0.51	0.70	0 – 3	--	--
Irresponsibility	0.86	0.89	0 – 3	--	--
Impulsivity	0.75	0.87	0 – 3	--	--
Distractibility	0.96	0.95	0 – 3	--	--
Risk Taking	0.47	0.73	0 – 3	--	--
Rigid Perfectionism	0.34	0.68	0 – 3	--	--
Unus. Beliefs/ Expers.	0.62	0.78	0 – 3	--	--
Eccentricity	0.28	0.62	0 – 3	--	--
Cog./ Percept. Dysfunc.	0.56	0.76	0 – 3	--	--

(table continues)

Supplemental Table S5 (cont.)

Variable	Mean	*SD*	Range	Cronbach's α / AIC	McDonald's ω
		DOMAINS CALCULATED FROM "BEST 3" FACETS			
Negative Affectivity	0.86	0.68	0–2.67	.68 / .41	.68
Detachment	0.71	0.68	0–2.67	.74 / .49	.76
Antagonism	0.54	0.65	0 – 3	.77 / .53	.79
Disinhibition	0.73	0.67	0–2.67	.68 / .41	.74
Psychoticism	0.49	0.59	0 – 3	.75 / .50	.76

Note. N=607. α = alpha. ω = omega. *SD* = Standard deviation. Unus. Beliefs/ Expers. = Unusual Beliefs and Experiences. Cog./ Percept. Dysfunc. = Cognitive and Perceptual Dysfunction. AIC = average interitem correlation.

[a]Not applicable
